# Ageing with Chagas disease: an overview of an urban Brazilian cohort in Rio de Janeiro

**DOI:** 10.1186/s13071-018-2929-y

**Published:** 2018-06-19

**Authors:** Alexandre Gomes Vizzoni, Margareth Catoia Varela, Luiz Henrique Conde Sangenis, Alejandro Marcel Hasslocher-Moreno, Pedro Emmanuel Alvarenga Americano do Brasil, Roberto Magalhães Saraiva

**Affiliations:** Evandro Chagas National Institute of Infectious Diseases, Oswaldo Cruz Foundation, Rio de Janeiro, Brazil

**Keywords:** Chagas disease, Elderly patients, Neglected tropical disease, *Trypanosoma cruzi*, Geoprocessing

## Abstract

**Background:**

Chagas disease control programmes have decreased the prevalence of Chagas disease in Latin America. Together with migration to urban areas and increase in life expectancy, a new scenario for Chagas disease has emerged in Brazil with most patients currently elderly individuals living in urban areas. However, acute Chagas disease cases still occur due to vector transmission by sylvatic vectors and oral transmission by contaminated food. Therefore, we characterized the clinical and epidemiological profile of the patients followed at Evandro Chagas National Institute of Infectious Diseases in Rio de Janeiro, Brazil. We aimed to identify the clinical forms, associated co-morbidities, and geographical areas where younger patients originate from. This will aid in the identification of potential challenges to be currently faced.

**Results:**

This is a cross-sectional study. Adult patients with chronic Chagas disease were recruited between March 2013 and April 2016. Clinical and epidemiological data were obtained from electronic medical records and interviews. The clinical form of the Chagas disease presented by the patients was determined following the Brazilian Consensus on Chagas disease. Six hundred and nineteen patients (mean age 60 ± 12 years; 56.9% women) were included in this study. Patients’ clinical forms were classified as follows: indeterminate 29.1%; cardiac 55.4%; digestive 5.5%; and mixed 10.0%. Patients aged over 65 years comprised 38% of the population. Hypertension was present in 347 (56%) patients, dyslipidemia in 261 patients (42%) and diabetes mellitus in 185 patients (30%). There were no differences regarding gender, race, comorbidities frequency or place of origin across Chagas disease clinical forms. Most of the elderly population originated from Bahia, Minas Gerais and Pernambuco states, while most of the younger patients were born in Ceará, Paraíba and Rio de Janeiro states.

**Conclusions:**

We described a great proportion of elderly patients in the composition of an urban Brazilian Chagas disease patient cohort with a high prevalence of comorbidities. We also identified a change in the pattern of the place of origin among younger patients.

**Electronic supplementary material:**

The online version of this article (10.1186/s13071-018-2929-y) contains supplementary material, which is available to authorized users.

## Background

Chagas disease (CD) or American trypanosomiasis is a potentially life-threatening illness caused by the parasite *Trypanosoma cruzi*. An estimated 5–6 million people live with CD in Latin American countries [1] and CD is responsible for approximately 12,000 deaths per year [2]. However, CD has been increasingly detected in the last decades in USA, Canada, many European and some Western Pacific countries as a result of migration from Latin America to the rest of the world [3, 4]. In Brazil, 1.1–4.6 million are estimated to live with chronic CD [1, 5, 6]. Between 2000 and 2011, CD was still the main cause of death due to neglected tropical diseases in Brazil with an average annual age-adjusted mortality of 3.4 deaths per 100,000 populations [7]. The economic burden is also high in Brazil which has the highest annual health-care costs in Latin America (mean $129,211,209) [8].

Chagas disease was largely confined to rural areas where wild triatomines adapted to human dwellings by creating domestic colonies in mud walls and roofs. However, the urbanization of Latin American countries led to an intense migration from rural to urban areas during the 20th century where the disease could still be transmitted by blood transfusion [9]. In Brazil, until the 1950s, CD was recognized as a rural endemic disease in a context of high social vulnerability and the main mode of transmission was vector-borne [6]. However, in the second half of the 20th century the Brazilian industrialization resulted in a great urbanization process with massive migration movements. In this period alone, the Brazilian urban population increased from 19 to 138 million inhabitants, growing on a 7.3-fold basis. Most of the urban demographic growth, between 1960 and 1980, was due to the intense rural-urban migratory flow with nearly 43 million people leaving the countryside toward the cities [10]. Therefore, CD posed an increasing health and economic burden to Latin American countries which was lastly recognized in the 1970s when extensive and coordinated control programmes started in Latin American countries, such as Brazil. In Brazil, 18 states were included in CD endemic areas with risk of disease transmission by the main vector *T. infestans* [11]. Systematic control measures led to a great decrease in houses domiciled by this vector and a simultaneous reduction in the transmission of *T. cruzi* to humans [5, 12]. Besides the control of triatomines domestic colonies, blood donor screening for CD also contributed to a dramatic decrease in the number of new CD cases. In 2006, Brazil received an international certification issued by the Pan American Health Organization (PAHO) and the World Health Organization for the interruption of CD transmission by blood transfusion and *T. infestans* [13].

Although the incidence of new CD cases has decreased, the number of individuals already infected with *T. cruzi* is still an issue of primary concern, as CD is a lifelong infection with a variable degree of morbidity. Almost one third of the *T. cruzi*-infected subjects will eventually develop chronic Chagas heart disease [6]. On the other hand, acute CD cases still occur linked to vector transmission by native vectors, which can occasionally invade domiciles. Acute cases may also occur through oral transmission, which is associated with food contaminated by infected vectors mainly in Amazon region [14].

The main consequence of CD control programmes over time is that most CD patients currently under health care are much older individuals when compared to subjects under health care in the 1990s [15, 16]. Together with the ageing of CD patients, the urbanization process of the Brazilian society has increased the probability of chronic-degenerative comorbidities occurring in patients with chronic CD [6]. Therefore, the aim of this study was to characterize the clinical and epidemiological profile of CD patients followed at the outpatient service of the Evandro Chagas National Institute of Infectious Disease (INI) located in the State of Rio de Janeiro, Brazil. This will aid in the identification of potential challenges to be faced to provide adequate healthcare to this elderly CD population and at the same time we will identify the epidemiological profile of young CD patients in order to identify the geographical areas from where they are originated.

## Methods

### Study design

This is a cross-sectional study conducted at INI from March 2013 to April 2016. Patients were recruited from patients attending the institutional outpatient service. The INI is a national reference center for treatment and research in infectious diseases and tropical medicine. All patients were previously diagnosed with 2 simultaneously positive CD serological tests (enzyme-linked immunosorbent assay and indirect immunofluorescence) [17].

The following data were obtained from electronic medical records filed at INI: gender, age, place of origin, comorbidities and symptoms related to CD, CD classification and left ventricular ejection fraction (LVEF). We considered the limit of 65 years-old in classifying patients as elderly, according to the World Health Organization [18].

The CD classification followed the Brazilian Consensus on Chagas disease: indeterminate form, cardiac form (stage A, B1, B2, C and D), digestive form (megaesophagus and megacolon) and mixed form (cardio digestive) [6].

### Geoprocessing methodology

The spatial analysis of health data was done in QGIS software version 2.12.2, using the map of the Brazilian municipal mesh of 2013. The naturalness of each patient was coded according to the Brazilian Institute of Geography and Statistics Municipality Codes table, which allowed the georeferencing in the maps used. We summed the number of cases per municipality and generated the maps of the dataset with the whole population, and stratified by age group.

### Statistical analysis

All statistical analyses were performed using SPSS version 21 (SPSS, Chicago, USA). All continuous values passed the Kolmogorov-Smirnov test of normality. Normally distributed data were described as mean ± standard deviation and compared by one-way analysis of variance (ANOVA) with Bonferroni correction. Categorical variables were described as absolute and percentage values and compared by means of contingency tables. A *P*-value of < 0.05 was considered statistically significant.

## Results

### Patients’ characteristics

From a total of 932 patients enrolled in our outpatient service during the period of the study, 670 (71.9%) were recruited and 619 patients (66.4%) agreed to participate in this study. There were a slightly higher number of women than men (56.9%). Women were slightly older than men (61.0 ± 11.9 years *vs* 59.0 ± 12.4 years, *t* = -2.05, *P* = 0.04). Regarding CD classification, 180 patients (29.1%) presented the indeterminate form, 343 (55.4%) presented the cardiac form, 34 (5.5%) the digestive form and 72 (10.0%) the mixed (cardio plus digestive) form. Patients with the cardiac form were further classified into stages: 116 (33.8%) belonged to stage A, 111 (32.4%) to stage B1, 19 (5.5%) to stage B2, 79 (23.0%) to stage C and 18 (5.3%) to stage D. Among patients with the digestive form, 29 (85.3%) presented megaesophagus and 5 patients (14.7%) presented megacolon. Among patients with the mixed form, most of them also presented megaesophagus (85.5%), while megacolon was present in 14.5% of the patients.

A total of 236 (38.1%) patients aged over 65 years-old, 309 (49.9%) aged between 45 and 64 years-old, and 74 (12.0%) aged under 45 years-old. There were no significant differences in gender or race distribution among the different CD clinical forms (Table 1). Regarding age, patients with the indeterminate form were younger than patients with the cardiac, digestive, or mixed forms (Table 1).

**Table 1:** Distribution of socio-demographic characteristics in the studied groups

Variable	Clinical form *n* (%) (*n* = 619)					
	Indeterminate (*n* = 180)	Cardiac (*n* = 343)	Digestive (*n* = 34)	Mixed (*n* = 62)	Total (*n*=619)	*P-value*
Sex						0.194
Male	76 (42.2)	156 (45.5)	9 (26.5)	26 (41.9)	267 (43.1)	–
Female	104 (57.8)	187 (54.5)	25 (73.5)	36 (58.1)	352 (56.9)	
Age, years	55.5 ± 12.7	61.5 ± 11.3	61.6 ± 13.9	65.2 ± 9.6	60.1 ± 12.1	< 0.001
Ethnicity						0.926
Caucasian	73 (40.5)	142 (41.4)	16 (47.1)	23 (36.5)	254 (41.0)	–
Afro-Brazilian	25 (13.9)	46 (13.4)	6 (17.6)	10 (15.9)	87 (14.1)	
Mixed/Pardo	82 (45.6)	155 (45.2)	12 (35.3)	29 (46.8)	278 (44.9)	
Place of origin						0.192
North	–	1 (0.3)	–	–	1 (0.2)	–
Northeast	118 (65.6)	243 (70.8)	24 (70.6)	40 (64.6)	425 (68.6)	
South	8 (4.4)	1 (0.3)	–	2 (3.2)	11 (1.8)	
Southeast	52 (28.9)	91 (26.6)	10 (29.4)	19 (30.6)	172 (27.8)	
Central-West	2 (1.1)	7 (2.0)	–	1 (1.6)	10 (1.6)	
Time outside the endemic area						
< 10 years	24 (13.8)	25 (7.5)	2 (6.1)	–	51 (8.5)	0.002
10–20 years	53 (30.5)	73 (22.0)	6 (18.2)	21 (35)	153 (25.5)	–
> 20 years	90 (51.7)	227 (68.4)	25 (75.7)	37 (61.7)	379 (63.3)	
Born in RJ state	7 (4.0)	7 (2.1)	–	2 (3.3)	16 (2.7)	

All data used for this study are described in detail in a supplemental dataset (Additional file 1: Table S1).

### Time living outside endemic area

The time living outside the endemic area was less than 10 years for 51 patients (8.5%), between 10 to 20 years for 153 patients (25.5%), and more than 20 years for 379 patients (63.3%). Additionally, 16 patients (2.7%) were born in the State of Rio de Janeiro and never lived in an endemic area. Therefore, most patients followed at our institution moved from rural to urban areas decades ago. There was a preponderance of people who moved away from endemic areas a longer time ago among patients with the cardiac (*χ*^2^ = 4.479, *df* = 3, *P* = 0.001) and mixed forms (*χ*^2^ = 9.434, *df* = 3, *P* = 0.009) than in patients with the indeterminate form.

### Place of birth

**Fig. 1:**
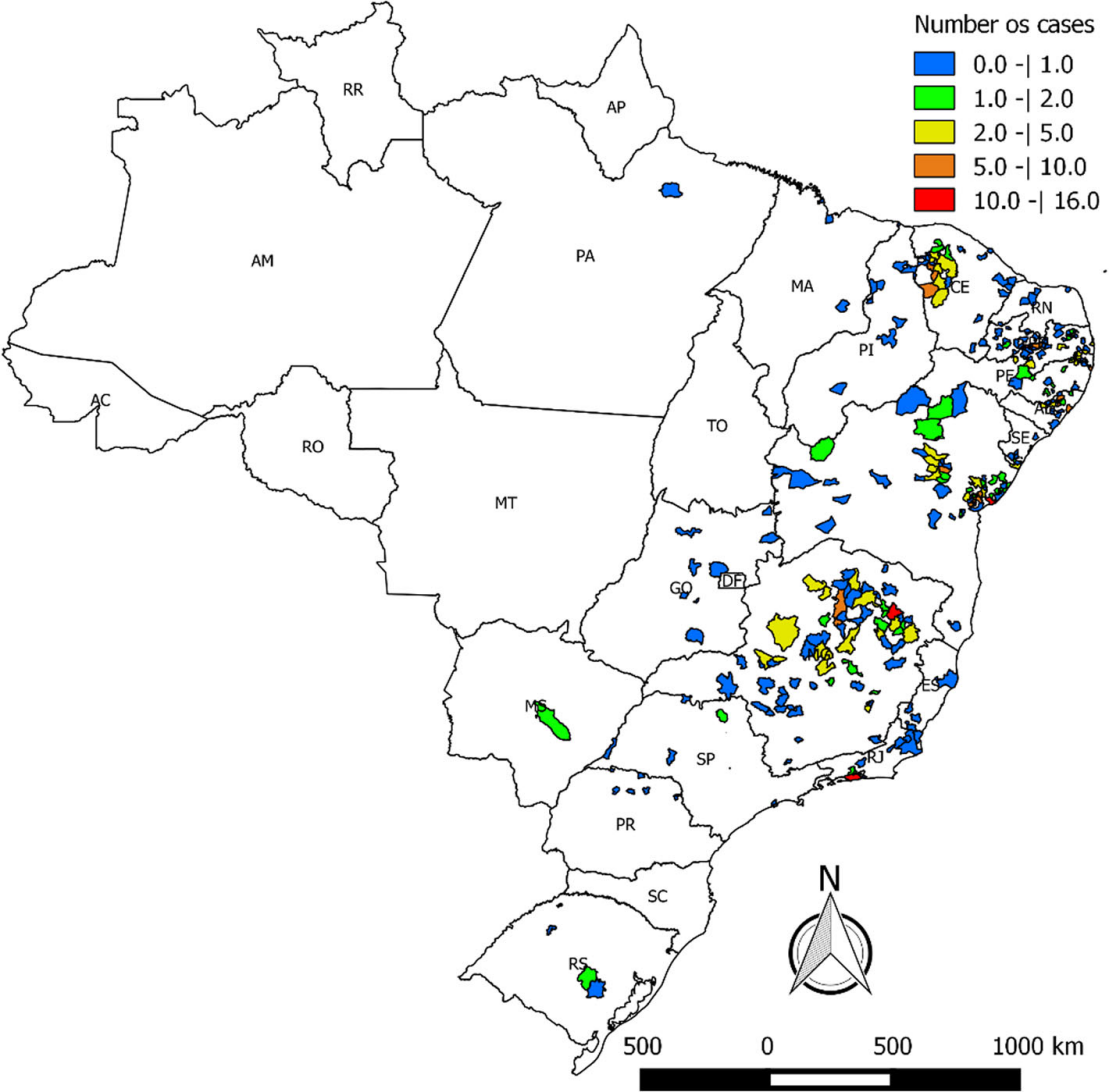
Place of origin of the patients with Chagas disease who constitute the INI cohort

Regarding place of origin, most patients were born in the Northeast (425, 68.6%) and Southeast (172, 27.8%) Brazilian regions. The number of patients from other Brazilian regions was lower: North (1, 0.2%), Central-West (10, 1.6%) and South (11, 1.8%) regions. The proportion of patients with the different CD forms was similar among Brazilian regions (Table 1, Fig. 1).

**Fig. 2:**
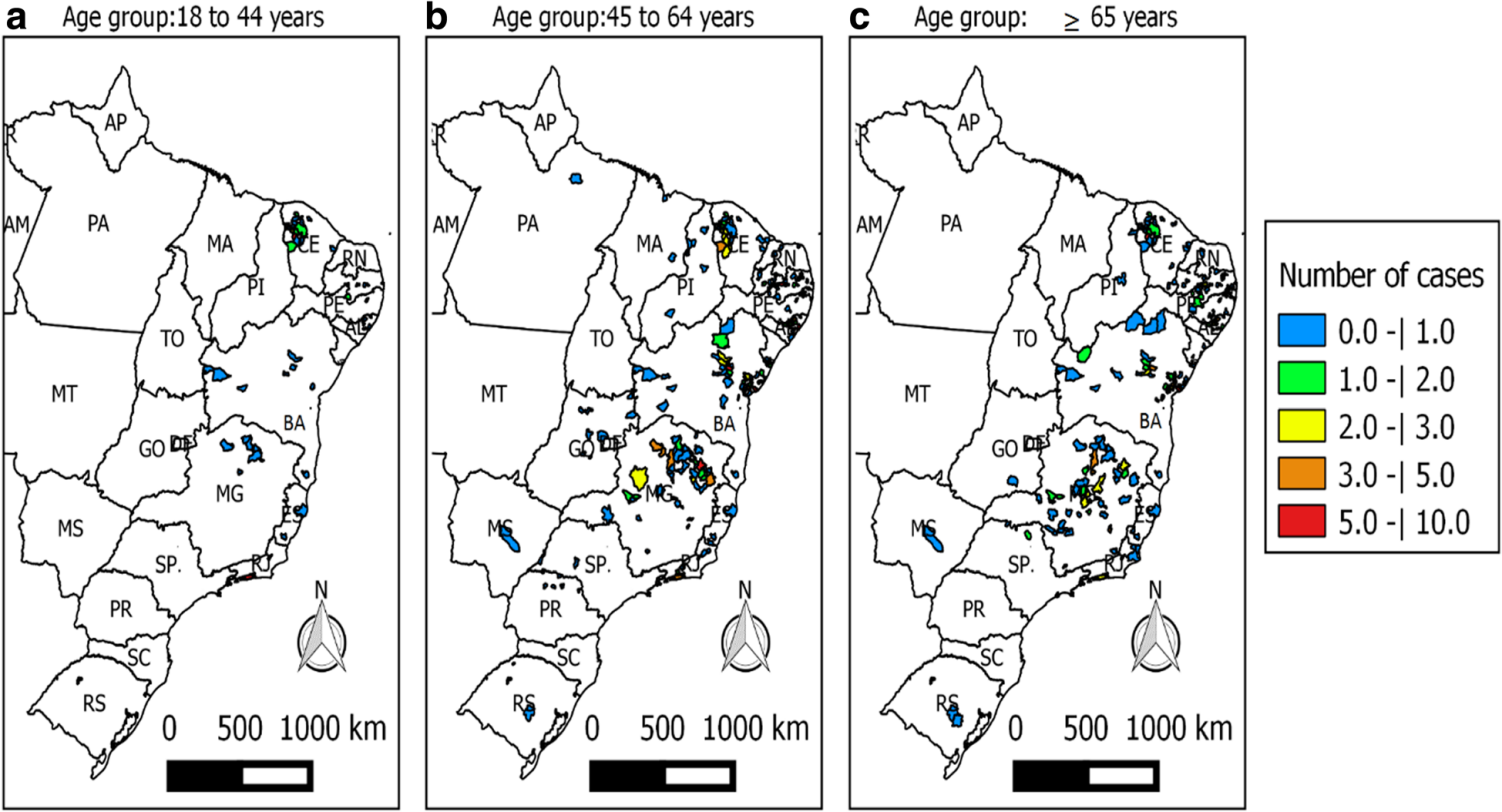
Spatial distribution of INI cohort Chagas disease patients stratified by age group (**a** 18–44 years-old, **b** 45–64 years-old, **c** ≥ 65 years-old)

Most patients came from rural areas. For instance, all patients from Minas Gerais State and 134 patients (89.3%) from the State of Bahia were born in rural areas. Among older individuals, most patients were born in Bahia, Minas Gerais and Pernambuco states. The municipalities most often cited by the patients as their places of origin were: Mairi (10, 6.6%), Cachoeira (7, 4.7%) and Feira de Santana (5, 3.3%) located in the State of Bahia, Araçuaí (11, 8.45%), Montes Claros (9, 6.9%) and Engenheiro Navarro (6, 4.6%) located in the State of Minas Gerais, and Timbaúba de Mocós (4, 6.5%) located in the State of Pernambuco. On the other hand, the majority of the younger individuals were born in Ceará, Paraíba e Rio de Janeiro states (*χ*^2^ =137.97, *df* = 34, *P* < 0.001). Among those, the most frequently cited municipalities were Nova Russas in the State of Ceará (*n* = 8), Desterro in the State of Paraíba (*n* = 7) and Rio de Janeiro in the State of Rio de Janeiro (*n* = 6; Table 2, Fig. 2).

**Table 2:** Distribution of patient frequencies by age group in the different states

State	Age group *n* (%)			
	18–44 years	45–64 years	≥65 years	Total
Alagoas	5 (6.8)	28 (9.1)	15 (6.3)	48 (7.8)
Bahia	7 (9.5)	72 (23.3)	71 (30.1)	150 (24.2)
Ceará	27 (36.5)	31 (10.0)	8 (3.9)	66 (10.7)
Espírito Santo	2 (2.7)	1 (0.3)	1 (0.4)	4 (0.6)
Goiás	–	7 (2.3)	1 (0.4)	8 (1.2)
Maranhão	–	3 (1.0)	–	3 (0.5)
Minas Gerais	6 (8.1)	65 (21.0)	59 (25.0)	130 (21.0)
Mato Grosso do Sul	–	1 (0.3)	1 (0.4)	2 (0.3)
Pará	–	1 (0.3)	–	1 (0.2)
Paraíba	14 (18.9)	40 (13.0)	24 (10.2)	78 (12.6)
Pernambuco	3 (4.0)	21 (6.8)	37 (15.6)	61 (9.9)
Piauí	–	7 (2.3)	2 (0.8)	9 (1.5)
Paraná	–	6 (2.0)	–	6 (1.0)
Rio de Janeiro	9 (12.2)	13 (4.2)	6 (2.5)	28 (4.5)
Rio Grande do Norte	–	5 (1.6)	1 (0.4)	6 (1.0)
Rio Grande do Sul	1 (1.3)	2 (0.6)	2 (0.8)	5 (0.8)
Sergipe		4 (1.3)	4 (1.6)	8 (1.2)
São Paulo		2 (0.6)	4 (1.6)	6 (1.0)

### Infection route

The route of transmission was vector-borne in 542 (90.5%) of the patients, blood transfusion in 10 patients (1.7%), vector-borne or blood transfusion in 23 patients (3.8%) and vertical in 24 patients (4.0%).

### Comorbidities and left ventricular ejection fraction

Hypertension was present in 347 patients (56.1%), dyslipidemia in 261 patients (42.2%), and type 2 diabetes mellitus in 185 patients (29.9%). Consequently, at least one comorbidity was present in 71.9% of the patients. Comorbidities frequency per clinical form was similar (Table 3), while hypertension, dyslipidemia and diabetes mellitus were all more prevalent among elderly patients (Table 4).

**Table 3:** Comorbidities frequencies according to Chagas disease clinical form

Comorbidities	Clinical form *n* (%)			
	Indeterminate (*n* = 180)	Cardiac (*n* = 343)	Digestive (*n* = 34)	Mixed (*n* = 62)
SAH	100 (55.5)	193 (56.2)	18 (52.9)	36 (58.0)
Dyslipidemia	84 (46.6)	137 (39.9)	12 (35.2)	28 (45.1)
Diabetes mellitus	53 (29.4)	112 (32.6)	5 (14.7)	15 (24.1)

*Abbreviation*: *SAH* systemic arterial hypertension

**Table 4:** Comorbidities frequencies according to age groups

Comorbidities	Age groups *n* (%)			*P*-value
	18–44 (*n* = 79)	45–64 (*n* = 309)	≥ 65 (*n* = 236)	
SAH	6 (7.6)	178 (57.6)	163 (69.1)	< 0.001
Dyslipidemia	14 (17.7)	138 (44.7)	109 (46.2)	< 0.001
Diabetes mellitus	6 (7.6)	93 (30.1)	86 (36.4)	< 0.001

Abbreviations: *SAH* systemic arterial hypertension

LVEF was lower in patients with the cardiac form (53.8 ± 16.3%) than in patients with the indeterminate (70.6 ± 6.4%) or digestive (68.1 ± 8.0%; *F* = 88.7, *P*<0.0001) forms. There was a progressive worsening of the LVEF from stage B1 to stage D of the cardiac form (Table 5).

**Table 5:** LVEF in patients with indeterminate and cardiac form of Chagas disease

	Stage	*n*	Mean	SD	*P*-value^a^
LVEF (%)	Indeterminate	164	70.6	6.4	
	A	112	69.2	7.5	
	B1	107	56.4	9.6*†	< 0.001
	B2	18	39.6	5.4*†‡	
	C	78	31.2	9.9*†‡	
	D	18	29.9	10.6*†‡#ǁ	

^a^One-way ANOVA Bonferroni *post-hoc *

*P < 0.05 *vs* indeterminate; †*P* < 0.05 *vs* stage A; ‡*P* < 0.05 *vs* stage B1; #*P* < 0.05 *vs* stage B2; ǁ*P* < 0.05 *vs* stage C

## Discussion

Chagas disease is considered one of the neglected tropical diseases (NTD) which affect around 1.4 billion people, mainly in the poorest countries. The agenda of Sustainable Development Goals (SDG) for 2030 proposed by the United Nations recognize that eradicating poverty is the greatest global challenge and an indispensable requirement for sustainable development. The insertion of a specific goal (goal number 3) for the combat to NTDs in the SDG agenda is highly relevant as recognizes the link between poverty and NTDs and that the combat to poverty and NTDs must come together [19]. Our results are very important in combating CD when we localize the geographical origin of patients living in urban areas. It is possible that the integration of reference services for CD throughout Brazil will allow the investigation if relatives of our patients living in other states present CD infection. It is also possible that the identification of the areas where our young patients were born will allow the investigation if those areas still present the conditions for active CD transmission.

The present study demonstrated a great proportion of elderly patients in the composition of an urban Brazilian CD patient cohort and a change in the most common place of birth among younger patients. Previous studies from our group described a mean age of the population followed at our outpatient service around 45.8 ± 11.7 years-old [20] . However, in the present study the mean age increased to 60 years-old in a period of 13 years. This finding is corroborated by others [21], which showed a progressive increase in the percentage of elderly patients enrolled in the Outpatient Unit of the Group for Studies into Chagas Disease at the Clinical Hospital of Campinas State University over 25 years (1980–2005). We also demonstrated a high frequency of comorbidities in our studied population with at least one comorbidity (hypertension, dyslipidemia or diabetes mellitus) present in 72% of the patients.

The increase in the proportion of elderly individuals among CD patients can be attributed to: a decrease in the CD incidence in Brazil due to the success of the vector and transfusion transmission control campaigns, improvement of the social status of the population with housing improvement in endemic regions, greater efficiency in diagnostic and therapeutic approaches [22, 23], and change in the Brazilian population general demographic profile with substantial aging of the population because of the fall in the rate of mortality associated with the rapid and marked decline of the fecundity rate. In fact, there is a progressive increase in the proportion of deaths among CD patients at a more advanced age, while the total number of deaths due to CD in Brazil is declining [24]. The increasing of migration and the progressive changes in rural economy modified the epidemiological patterns of the disease, mainly in terms of its transmission and medical attention. Several areas have been modernized in terms of housing and production aspects with a radical change from the classical subsistence way of life to an agroindustrial and large-scale economy [12, 25].

The actions taken to control CD transmission in South America aimed at improving housing in endemic areas for CD and fulfilling the objectives of the Southern Cone Initiative [26] (of which Brazil is one the signatories), which consisted of eliminating *T. infestans* infestations from houses and peridomestic environments in endemic areas, reducing and eliminating the infestation of other triatomine species in the same areas occupied by *T. infestans* and reducing and eliminating the transmission by blood transfusions [26, 27]. Other factors also contributed to decrease the incidence of CD in Brazil, such as the urbanization process of the country with intense migration from rural to urban areas observed since the 1940s, and the increase in the income of the population [28].

The places of birth distribution of our cohort reflect traditional endemic areas [6]. As the route of transmission of more than 90% of our patients was vector-borne, we consider that the place of birth was the place where most patients were infected. Time living outside endemic areas is longer among patients with the cardiac and mixed forms than patients with the indeterminate form. This probably reflects longer time of disease among patients with determined forms of the disease than in patients whose disease had not yet progressed. However, the place of birth differs between older and younger patients probably due to different vector species present in the areas where younger patients were born. Therefore, the effectiveness of the vector control measures undertaken is probably different due to the different characteristics of the triatomines present in these areas. The main vector responsible for vectorial transmission in Bahia and Minas Gerais states was *T. infestans*, which was eradicated from domiciles, while *T. infestans* is not found in the State of Ceará where the main vector is the *Triatoma brasiliensis.* In the State of Paraíba, *T. brasiliensis* and *Triatoma pseudomaculata* are highly prevalent. Both species are native and present in the Caatinga ecoregion of both Ceará and Paraíba states [29, 30]. *Triatoma pseudomaculata* is considered a vector with an intermediate capacity to transmit *T. cruzi* while *T. brasiliensis* has a high effectiveness for CD transmission and is currently the most important vector in the Northeast Brazilian region [29, 30] . These native triatomines need other control measures to avoid their role in the *T. cruzi* transmission. These control measures should focus on barriers to house infestation, such as indoor insecticide spraying and/or housing improvement interventions aimed at reducing the suitability of the domestic habitat [31–33]. The third state with more cases among younger individuals was Rio de Janeiro, the state where our institution is located. This can be explained mostly by vertical transmission and also by autochthonous transmission by *T. vitticeps* in specific Rio de Janeiro State rural areas [34]. An alternative explanation for the difference in place of birth present among different age groups would be a change in migration flow pattern from the Northeast Brazilian region to the State of Rio de Janeiro. In fact, during the 1970s and 1980s there was an intense flow from the Northeast region and State of Minas Gerais to the Southeast Brazilian region attracted by the economic development present in urban areas at that time, mainly in São Paulo and Rio de Janeiro states [10]. Nowadays, the economic development of the Northeast region associated to social policies contributes to a change in migration flow with a decrease in interstate migration flow and an increase in return migration. These dynamic changes in migration flow patterns may also contribute to the difference found in the place of birth composition between older and younger CD patients [35].

Our study and others [36, 37] have shown a higher proportion of women with CD, although *T. cruzi* infection does not have a gender predilection [38]. This difference may be related to the more frequent use of health services by women, even after controlling for restrictions in routine activities due to health reasons [39]. The predominant clinical form of CD among patients included in this study was the cardiac form, followed by the indeterminate form, while digestive and mixed forms represented only 15% of the cohort. These findings are consistent with other studies in the literature, where the cardiac form is prevalent among elderly patients with CD [16, 40]. However, asymptomatic patients may be unaware of their condition and may be not followed at health services. This would increase the proportion of patients with cardiac form in the cohort followed by health services.

Comorbidities frequency among elderly patients with CD in our study was high. The frequency of arterial hypertension among elderly patients with CD in our study was similar to others, however, diabetes mellitus and dyslipidemia frequencies in our study was higher than those described by others [40, 41]. While we described diabetes mellitus and dyslipidemia frequencies of 36 and 46%, respectively, others described diabetes mellitus frequency ranging between 10–14.4% [40, 41], and dyslipidemia frequency ranging between 20–31.9% [40, 41]. Arterial hypertension frequency among elderly patients with CD in our study was higher than in Brazilian elderly population. According to Andrade et al. [42], the frequency of hypertension in the general elderly population was 52.7–55.0% while in our study hypertension frequency was 69%. Dyslipidemia and diabetes mellitus were also more frequent in our study than in the general Brazilian population. In the study of Iser et al. [43], the frequency of diabetes mellitus in general population was 19.9% and Bos et al. [44] reported dyslipidemia in 14.5% of the Brazilian general population. These differences may be accounted for the fact that people with comorbidities seek medical attention more frequently than people without comorbidities.

## Conclusions

We described a great proportion of elderly patients in the composition of an urban Brazilian CD patient cohort and a change in the pattern of the place of origin among younger patients. We also demonstrated a high frequency of comorbidities in our studied population. In fact, the elderly patients with CD comprise a group that has a high frequency of systemic arterial hypertension and other comorbidities (diabetes mellitus and dyslipidemia). Those are challenges to be overcome when developing new public health policies to address CD.

## Additional file

Additional file 1: **Table S1.** Dataset describing the raw data that support the conclusions of this article. (XLSX 55 kb)

## Abbreviations

ANOVA: Analysis of variance
CD: Chagas disease
INI: Evandro Chagas National Institute of Infectious Disease
LVEF: Left ventricular ejection fraction
PAHO: Pan American Health Organization
SAH: Systemic arterial hypertension
